# Alterations in Metabolic Status and Headshaking Behavior Following Intravenous Administration of Hypertonic Solutions in Horses with Trigeminal-Mediated Headshaking

**DOI:** 10.3390/ani8070102

**Published:** 2018-06-25

**Authors:** Shara Sheldon, Monica Aleman, Lais Costa, A. Cristina Santoyo, Quinn Howey, John Madigan

**Affiliations:** 1 Department of Nutrition, University of California, Davis, CA 95616, USA; sasheldon@ucdavis.edu; 2 Department of Medicine and Epidemiology, School of Veterinary Medicine, University of California, Davis, CA 95616, USA; lrcosta@ucdavis.edu (L.C.); mvzanasantoyo@gmail.com(A.C.S.); qhowey@gmail.com (Q.H.); jemadigan@ucdavis.edu (J.M.)

**Keywords:** equine, trigeminal, headshaking, hypertonic saline, hypertonic sodium bicarbonate, intravenous, metabolic, pH, horses

## Abstract

**Simple Summary:**

Horses with trigeminal-mediated headshaking syndrome suffer from pain and electric-shock-like sensation in the nerve that runs across their faces (trigeminal nerve), leading to violent head jerking that impairs their performance and quality of life. This condition has no curative treatments and often leads to euthanasia of the animal. Changes in blood components (pH, electrolytes) are known to affect nerve pain. To investigate this, three different kinds of fluids (with varying pH and electrolytes) were given in the vein to horses affected with trigeminal-mediated headshaking. The headshaking behaviors and changes in blood composition were assessed after each treatment. Changes in blood composition were transient, and there was a greater than 50% decrease in headshakes/minute with a high pH treatment. The limited effects following these fluids were likely due to normal mechanisms of regulation of blood levels of these salts and minerals. Further investigations of changes in electrolytes that might affect nerve firing should be explored.

**Abstract:**

Trigeminal-nerve-mediated headshaking represents a major welfare challenge for owners and veterinarians and is caused by a low threshold firing of the trigeminal nerve resulting in pain manifested as violent head jerking that often terminates the horse’s career and life due to poor quality of life and suffering. As metabolic changes such as acid–base status and electrolytes play a role in nerve firing, this study sought to assess the effects following administration of hypertonic solutions on headshaking behavior in affected horses. This prospective randomized controlled crossover design utilized six horses affected with trigeminal-mediated headshaking and three treatment groups receiving intravenous administration of 5% dextrose solution at 2 mL/kg bwt (DS), NaCl 7.5% at 4 mL/kg bwt (HS), or NaHCO_3_ 8.4% at 2 mmol/kg bwt (HB). Horses were assessed for headshaking behavior changes at times T0 (baseline, before infusion) and T15, 30, 60, 120 min post infusion. Venous blood variables: pH, HCO_3_^−^, standard base excess (SBE), Na^+^, Cl^−^, K^+^, Ca^2+^, Mg^2+^, total magnesium (tMg), glucose, and lactate were measured at T0 (baseline, before infusion) and T5, 15, 30, 60, 120 min post infusion. Strong ion difference (SID) and anion gap (AG) were calculated for each time point. With HB treatment, there was greater than 50% reduction in headshaking rate. There was an effect of time on increasing headshaking rate. There was an effect of breed on headshaking rate. Changes in blood parameters following DS were virtually absent. Infusion of HS caused mild changes and did not vary much from baseline except for SID and AG. Only infusion of HB caused blood pH and HCO_3_^−^ to be outside of the physiologic range (alkalemia and metabolic alkalosis, respectively), SBE to double or triple, AG to decrease, and SID to increase compared to baseline. Infusion of DS was followed by increase in blood glucose and decrease in blood Na^+^. Infusion of HS was followed by increase in Na^+^ and Cl^−^ and decrease in Mg^2+^. Infusion of HB was followed by decrease in Mg^2+^. Blood tMg, K^+^, and Ca^2+^ decreased slightly, but did not vary greatly from baseline following any of the treatments, remaining within physiologic ranges. Changes in blood composition were transient. Among all treatments, only HB had an effect on headshaking rate. The limited effects following these fluids were likely due to normal mechanisms of regulation of blood levels of pH and electrolytes. Further investigations of changes in electrolytes that might affect nerve firing should be explored.

## 1. Introduction

Idiopathic or trigeminal-mediated headshaking in horses is a debilitating and painful condition that can seriously compromise athletic performance, daily life activities, and quality of life, representing a major welfare problem that often results in euthanasia [1]. Although the condition has been described in the literature for over one hundred years, its pathogenesis remains poorly understood [2]. The clinical signs consist of sudden violent head shakes, nose itching and rubbing, snorting, striking their face with their front limbs, burning, tingling, or electric-like sensations that fit the description for neuropathic pain [3,4,5]. The behavior and anxious facial expression of the horse appear to be that of stress. Indeed, studies on somatosensory nerve conduction of the trigeminal nerve in horses with idiopathic headshaking demonstrated lower threshold to trigger nerve conduction (10 times lower) of the maxillary branch of the trigeminal nerve in affected horses compared to control horses [6,7]. These findings suggested a functional abnormality and led to renaming the condition as trigeminal-mediated headshaking, a term that differentiates it from other causes of headshaking [1,6,7]. Furthermore, the low threshold for firing of the nerve explains why sometimes apparently innocuous stimuli such as light, sound, wind, or touch might trigger apparent intense pain [7]. Other times, the signs appear spontaneously [7]. The disorder is seen primarily in geldings with an age onset of about 8 to 9 years, and it has a seasonal component, happening more often in the spring and summer months [3]. A number of treatment options have been reported with inconsistent results [1,8,9,10,11,12].

The cause of trigeminal-mediated headshaking is unknown, however, a multifactorial etiology is suspected because 60% of affected horses present exacerbation of clinical signs in a seasonal fashion (mainly spring and summer months) and geldings are over-represented (72%) [1]. These factors may include environmental, dietary, and hormonal contributions. Dietary component might be associated with seasonality, as the switch of forage from a winter crop to a spring crop coincides with onset of exacerbation of signs. Dietary components, including electrolytes and mineral content, in forage are known to change with season, soil, and harvest management [13,14,15]. Moreover, it has been shown that changes in blood pH and electrolytes, especially calcium and magnesium, affect nerve conduction [16,17,18]. The current study was implemented to evaluate the potential effects of experimental changes in blood pH and electrolytes on headshaking [16]. This preliminary study sought to utilize horses diagnosed with trigeminal-mediated headshaking syndrome and assess the effects of intravenous infusion of 5% dextrose, hypertonic saline, and hypertonic sodium bicarbonate solutions on the frequency of headshaking. We hypothesized that alterations in blood pH and electrolytes will affect headshaking behavior.

## 2. Materials and Methods

### 2.1. Subjects and Facilities

This study included a total of six male castrated horses with naturally-occurring headshaking that were donated to the Center for Equine Health at University of California, Davis, CA, USA. The inclusion criteria consisted of fulfillment of the diagnosis of trigeminal-mediated headshaking by exclusion of all other causes of headshaking. At the time of donation, horses received all recommended vaccinations and deworming per Center for Equine Health protocols. Prior to entering the study, all horses underwent a thorough physical and neurologic examination performed by a board-certified, large-animal internist and neurologist (Monica Aleman) followed by a detailed diagnostic workup. Diagnostic workup included oral, ophthalmic and otoscopic examinations, complete cell blood count, serum biochemical profile, skull radiographs, and upper airway endoscopy. Breeds of enrolled horses included Quarter Horse breeds (*n* = 4) and Thoroughbreds (*n* = 2), ages 5 to 13 years, weighing 472–580 kg. The horses were housed in covered box stalls bedded with wood shavings, having free access to fresh water (automatic waterer), and fed twice daily a hay diet consisting of grass-alfalfa mix with a dietary cation–anion balance (DCAB = [Na^+^ + K^+^] − [Cl^−^ + SO_4_^−^]) of 31 mEq/100g.

### 2.2. Experimental Design

The study was a randomized controlled crossover (3 by 3) experimental design, where each horse served as its own control. The horses were randomized to one of the three treatment groups with a washout period greater than a week between treatments. All horses received all three treatments. Intravenous catheters were placed in the left jugular vein under aseptic technique. Horses received sterile fluids intravenously: Dextrose Solution (DS) group received 5% dextrose solution at 2 mL/kg bwt, Hypertonic Saline (HS) group received NaCl 7.5% solution at 4 mL/kg bwt, and Hypertonic Sodium Bicarbonate (HB) group received NaHCO_3_ 8.4% solution at 2 mmol/kg bwt. The infusion time was 30 min, and after infusion of treatment, the catheters were removed.

### 2.3. Sample Collection

Heparinized blood samples were collected by venipuncture from the right jugular vein at times 0 (baseline, before infusion) and 5, 15, 30, 60, 120 min post infusion and placed on ice immediately.

### 2.4. Blood Analysis

Venous blood pH, standard base excess (SBE), HCO_3_^−^, Na^+^, Cl^−^, K^+^, Ca^2+^, glucose, and lactate in heparinized blood samples were determined using an ABL815 FLEX (Radiometer America Inc., Brea, CA, USA). Total magnesium (tMg) and ionized magnesium (Mg^2+^) in heparinized blood samples were determined using NOVA 8 (NOVA Biomedical, Waltham, MA, USA). The strong ion difference (SID) was calculated using the Stewart equation as follows: [(Na^+^ + K^+^ + Ca^2+^ + Mg^2+^) − (Cl^−^)] [19]. Anion Gap (AG) was calculated using the equation as follows: [(Na^+^ + K^+^) − (Cl^−^ + HCO_3_^−^)] [19].

### 2.5. Behavioral Analysis

Horses were placed in individual round pens, without tack or halters, and evaluated by three independent, trained evaluators for headshaking behavior while at a walk (1 min), trot (1–3 min), canter (1 min), and walk again (1 min) at 5 time points (i.e., T0 (baseline, before infusion) and T15, 30, 60, 120 min post infusion). After each time point, the horses were returned to their immediately adjacent stalls. Evaluators were unaware of which treatment each horse received on any particular day. The headshaking behavior (including headshakes/minute, head tossing/minute, nose rubbing/minute, dropped head/minute, and snorting/minute) was recorded for each horse during each level of exercise at each time point by the three evaluators. A median of each headshaking behavior per minute for each horse at each time point during each level of exercise was obtained. Variability between evaluators was determined.

### 2.6. Statistical Analysis

Data were analyzed using Stata Statistical Software, Release 14, StataCorp LP 2015, College Station, TX. A multilevel mixed-effects Poisson regression model examined the main and interactive fixed effects of treatment groups (DS, HS, or HB), time period 0 (baseline), and 15, 30, 60, 120 min post infusion, period, and breed, with individual horses as the random effect, on headshaking. Each period (walk, trot, canter, walk) was evaluated separately. Within each period, a model was created to evaluate the possible interaction between treatment and time; if the interaction was significant, two further sets of analyses (effect of treatment at individual times, effect of time within individual treatments) were performed. If the interaction was not significant, then a main effects-only model was fit. Results are presented as incidence rate ratios (IRR), *p* values, and 95% confidence intervals (CI). A color-coded dot plot with the regression line was used to display longitudinal data for individual horses.

A multilevel mixed-effects analysis of variance model was also used to look at the main and interactive effects of treatment groups (DS, HS, or HB) and time period (baseline or 0, and 5, 15, 30, 60, 120 min post infusion), with individual horse as random effect, on blood parameters (pH, K^+^, Na^+^, Cl^−^, SBE, HCO_3_^−^, Ca^2+^, glucose, lactate, tMg, Mg^2+^) and calculated values of SID and AG. Each period (walk, trot, canter, walk) was evaluated separately. Within each period, a model was created to evaluate the possible interaction between treatment and time; if the interaction was significant, two further sets of analyses (effect of treatment at individual times, effect of time within individual treatments) were performed. If the interaction was not significant, then a main effects-only model was fit.

### 2.7. Ethical Approval

Ethical approval was provided by UC Davis IACUC on 24 March 2015. This institution is accredited by the Association for Assessment and Accreditation of Laboratory Animal Care, International (AAALAC). This institution has an Animal Welfare Assurance on file with the Office of Laboratory Animal Welfare (OLAW). The Assurance Number is A3433-01.

## 3. Results

### 3.1. Behavior Results

**Figure 1:**
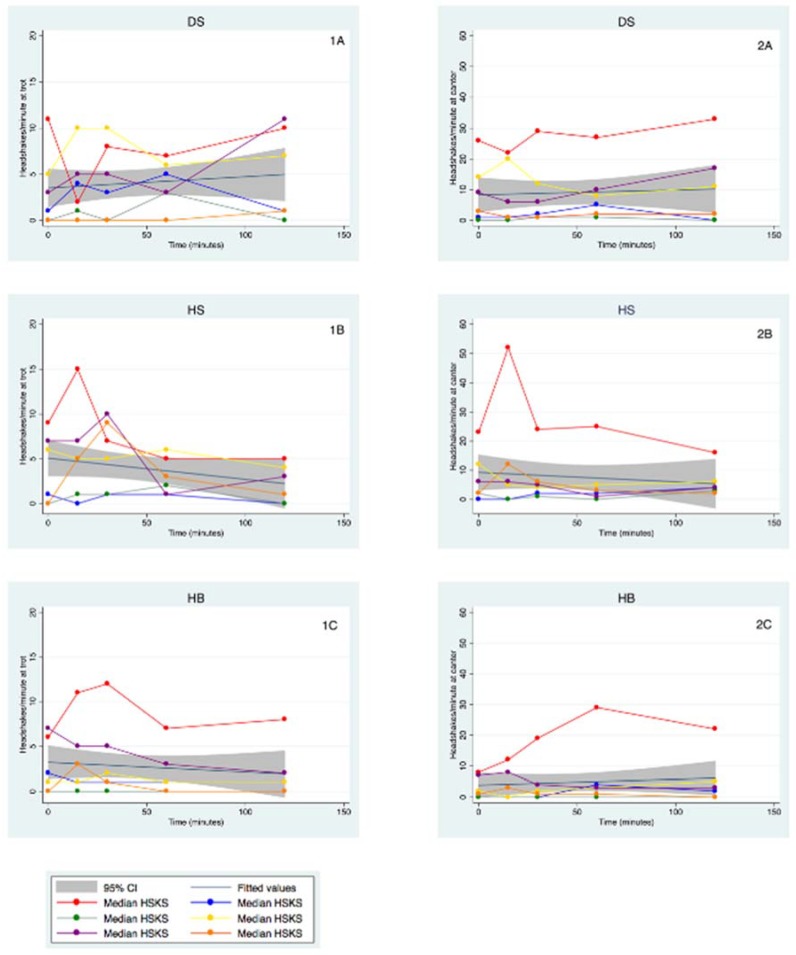
Headshaking behavior for individual horses and overall change over time (Graphs **1A**–**C**) at the trot; (Graphs **2A**–**C**) at the canter. Each horse is displayed as a different color and the overall change in headshaking behavior is represented by the regression line. Top graphs (**1A** and **2A**) = DS (5% dextrose solution at 2 mL/kg bwt), middle graphs (**1B** and **2B**) = HS (NaCl 7.5% solution at 4 mL/kg bwt), and bottom graphs (**1C** and **2C**) = HB (NaHCO_3_ 8.4% solution at 2 mmol/kg bwt).

The headshaking behavior, which included headshakes/minute, head tossing/minute, nose rubbing/minute, dropped head/minute and snorting/minute, varied widely among horses. The overall median and interquartile range (IQR) for each headshaking behavior for individual horses at the trot and canter were assessed (Table 1). The variance amongst scorer was not statistically significant (data not shown). For the statistical analysis, we focused on the headshakes/minute as this was the most important and consistent behavior of affected horses. The range of headshakes/minute between horses varied widely, as some horses were very severely affected (e.g., baseline at trot 11 headshakes/min and at canter 23 headshakes/min) whereas others were not (Table 1). Individual horses responded differently for each solution (Table 1). Figure 1 depicts the longitudinal data for individual horses, indicating changes in median headshakes/minute for each horse (color-coded) and the overall change (regression line) over time, for each treatment group DS (top graph), HB (middle graph), and HS (bottom graph), at the trot (left) and at the canter (right).

A mixed-effects Poisson regression model evaluated the effects of treatment and time while controlling for breed on median headshakes/minute. There was an effect of HB treatment controlling for all breeds, times, and periods (walk, trot, canter, walk) with a 58% reduction in median headshakes/minute when compared to DS (0.579 IRR, C.I. [0.49, 0.67], *p* < 0.001, Table 2). When restricted to the trot period, there was a 67% reduction in median headshakes/minute for HB treatment compared to DS (0.672 IRR, C.I. [0.51, 0.89], *p* = 0.005, Table 2). When restricted to the canter period, there was a 52% reduction in median headshakes/minute with HB treatment compared to DS (0.522 IRR, C.I. [0.43, 0.64], *p* < 0.001, Table 2). There was an effect of time at T15 compared to T0 controlling for all periods, treatments, and breed, with a 25% increase in median headshakes/minute (1.25 IRR, C.I. [1.04, 1.51], *p* = 0.020). There were no effects of time when the model was restricted to the trot period and HS treatment. There was an effect of time when the model was restricted to the canter period and HS treatment, with T15 having a 70% increase in median headshakes/minute (1.67 IRR, C.I. [1.15, 2.41], *p* = 0.007, Table 3) compared to T0. There was an effect of time when the model was restricted to the canter period and HB treatment, with a twofold increase in median headshakes/minute at T60 (2.00 IRR, C.I. [1.17, 3.42], *p* = 0.011, Table 3) compared to T0. There was an effect of breed, with Thoroughbreds having a 9% reduction in median headshakes/minute when compared to Quarter Horse breeds when the model was restricted to the trot (0.09 IRR, C.I. [0.02, 0.32], *p* < 0.001), and Thoroughbreds having a 10% reduction in median headshakes/minute when compared to Quarter Horse breeds when the model was restricted to the canter (0.01 IRR, C.I. [0.01, 0.50], *p* = 0.005).

There was a significant interaction between treatment and time when the model was restricted to the canter period, with HB and T60, with a twofold increase in median headshakes/minute (2.00 IRR, *p* = 0.039, C.I. [1.04, 3.86]) as compared to DS and T0, and with HS and T15, a 76% increase in median headshakes/minute (1.76 IRR, C.I. [1.04, 3.01], *p* = 0.037). There was a significant interaction between treatment and time when the model was restricted to the canter period, with HS and T120 having a 37% reduction in median headshakes/minute (0.37 IRR, C.I. [0.155, 0.91], *p* = 0.031) as compared to DS and T0. Table 4 depicts the IRR of treatment and breed and period on headshaking. There was a significant interaction between treatment and breed when the model was restricted to the trot period, with HS and Thoroughbreds having a fivefold increase in median headshakes/minute (5.2 IRR, C.I. [1.91, 14.38], *p* = 0.001) when compared to DS and Quarter Horse breeds. There was a significant interaction between treatment and breed when the model was restricted to the canter, with HS and Thoroughbreds having a threefold increase in headshakes/minute (3.61 IRR, C.I. [1.77, 7.36], *p* < 0.001) when compared to DS and Quarter Horse breeds. 

### 3.2. Blood Results

At baseline, all blood parameters measured were within reference range except SBE, which was uniformly above the reference range (mean 6.1 ± 0.3 mmol/L for DS, 4.8 ± 0.2 for HS, 5.4 ± 0.3 for HB, Table 5). Therefore, comparisons for SBE were made from baseline values. Overall, changes in blood pH, HCO_3_^−^, SBE, SID, and AG following DS were virtually absent (Table 5 and Table 6). Infusion of HS led to mild changes in blood pH, SBE, SID, and AG which did not vary much from baseline (Table 4 and Table 5). Only infusion of HB caused blood pH and HCO_3_^−^ and SID to be outside of the physiologic range (alkalemia and metabolic alkalosis, respectively), and SBE to double or triple compared to baseline, as indicated in Table 5, and AG to further decrease from baseline (Table 6).

**Figure 2:**
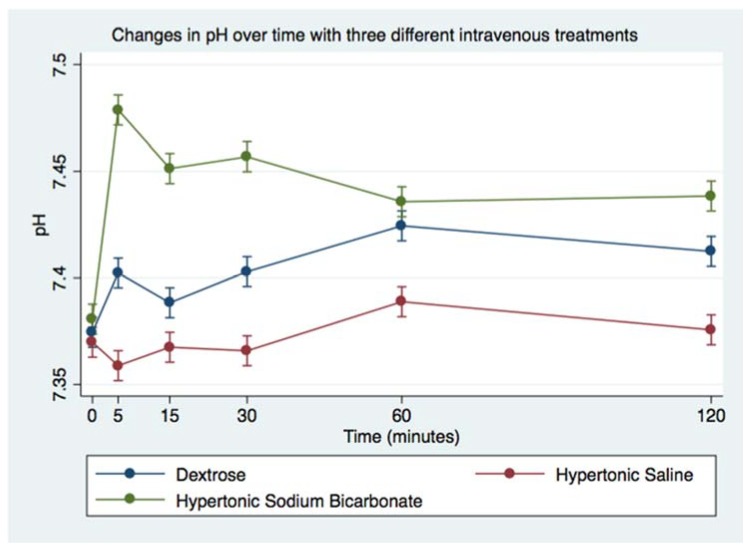
Overall effect of treatment on venous blood pH over time. The mean and SEM venous blood pH for all six horses. Blue = Treatment Group DS (5% dextrose solution at 2 mL/kg bwt); Green = Treatment Group HB (NaHCO_3_ 8.4% solution at 2 mmol/kg bwt); and Red = Treatment Group HS (NaCl 7.5% solution at 4 mL/kg bwt).

**Figure 3:**
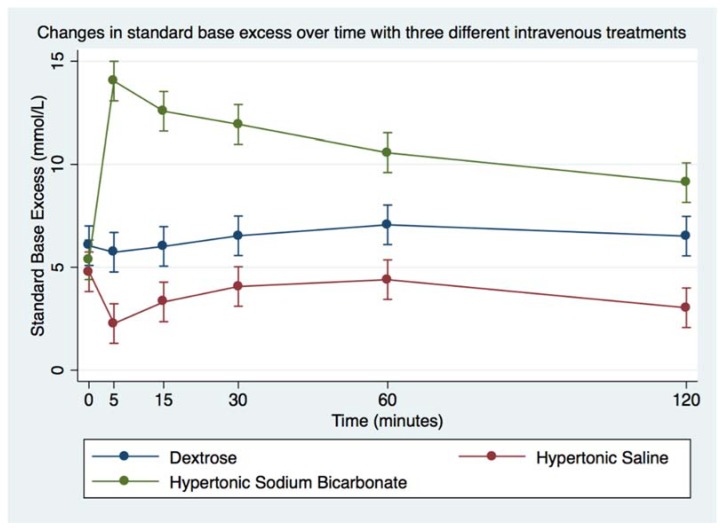
Overall effect of treatment on the blood SBE over time. The mean and SEM venous blood SBE for all six horses. Blue = Treatment Group DS (5% dextrose solution at 2 mL/kg bwt); Green = Treatment Group HB (NaHCO_3_ 8.4% solution at 2 mmol/kg bwt); and Red = Treatment Group HS (NaCl 7.5% solution at 4 mL/kg bwt).

**Figure 4:**
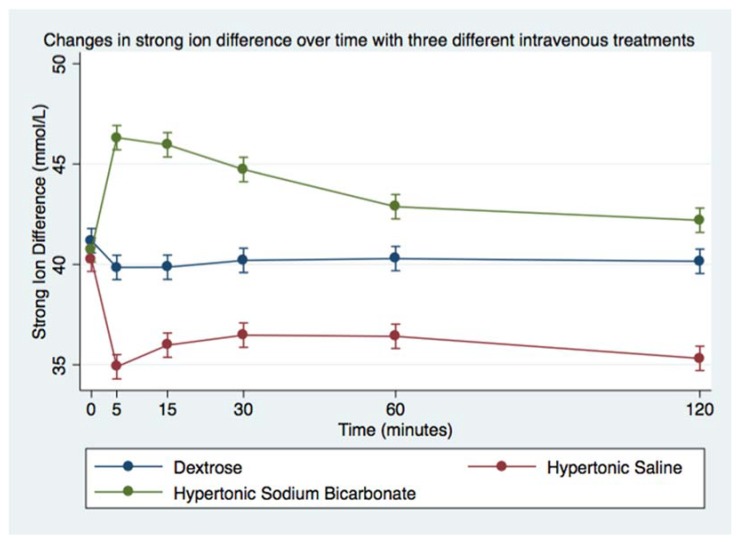
Overall effect of treatment on the blood SID over time. The mean and SEM venous blood SID for all six horses. Blue = Treatment Group DS (5% dextrose solution at 2 mL/kg bwt); Green = Treatment Group HB (NaHCO_3_ 8.4% solution at 2 mmol/kg bwt); and Red = Treatment Group HS (NaCl 7.5% solution at 4 mL/kg bwt).

**Figure 5:**
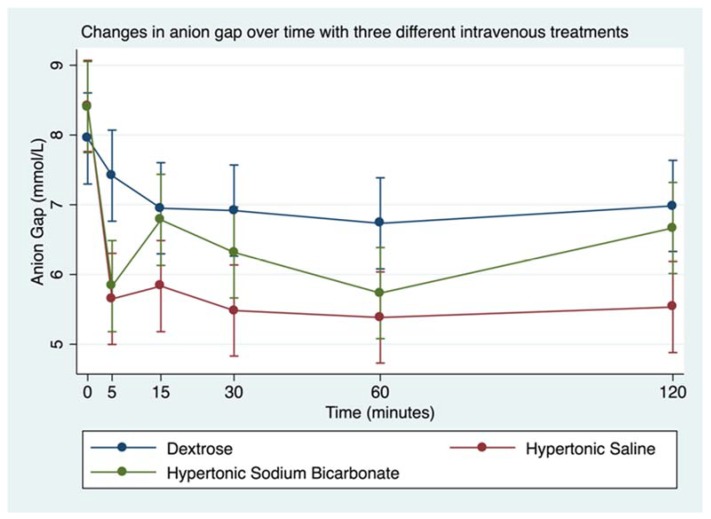
Overall effect of treatment on the blood AG over time. The mean and SEM venous blood AG for all six horses. Blue = Treatment Group DS (5% dextrose solution at 2 mL/kg bwt); Green = Treatment Group HB (NaHCO_3_ 8.4% solution at 2 mmol/kg bwt); and Red = Treatment Group HS (NaCl 7.5% solution at 4 mL/kg bwt).

Infusion of HS led to minimal decrease in blood pH, whereas infusion of HB led to significant increase in blood pH (Figure 2 and Table 5). Following infusion of HS, there was a decrease in blood SBE (from mean 4.8 ± 0.2 to 2.3 ± 0.2 mmol/L), whereas infusion of HB led to significant increase in blood SBE (from mean 5.4 ± 0.3 to 14 ± 0.4 mmol/L; Figure 3 and Table 5). Additionally, SID changed after both hypertonic treatments; HS caused SID to decrease below reference range whereas HB caused SID to rise above reference range (Table 6, Figure 4). In contrast, AG was uniformly below reference range at baseline (mean 8 + 0 mmol/L for DS, 8 + 0 mmol/L for HS, 8 + 1 mmol/L for HB, Table 6, Figure 5), and it did not change much after infusion of any of the treatments. 

**Figure 6:**
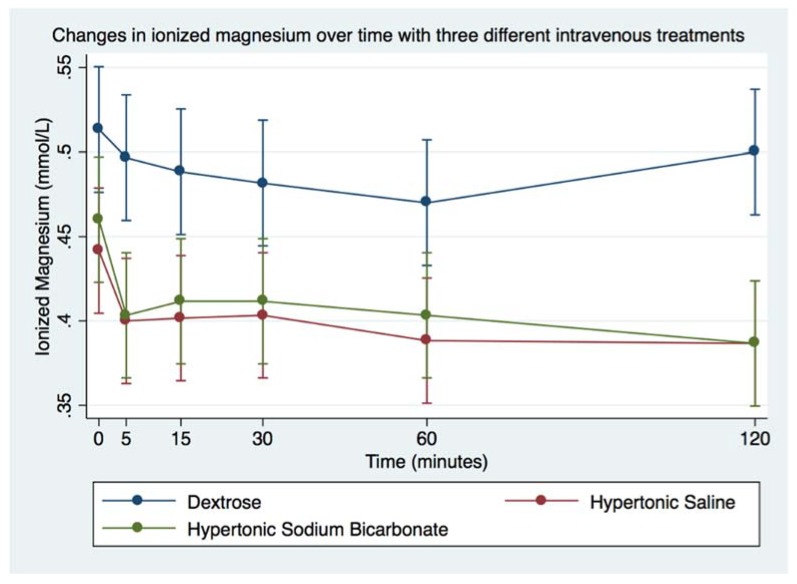
Overall effect of treatment on the blood ionized magnesium (Mg^2+^) over time. The mean and SEM venous blood Mg^2+^ for all six horses. Blue = Treatment Group DS (5% dextrose solution at 2 mL/kg bwt); Green = Treatment Group HB (NaHCO_3_ 8.4% solution at 2 mmol/kg bwt); and Red = Treatment Group HS (NaCl 7.5% solution at 4 mL/kg bwt).

**Figure 7:**
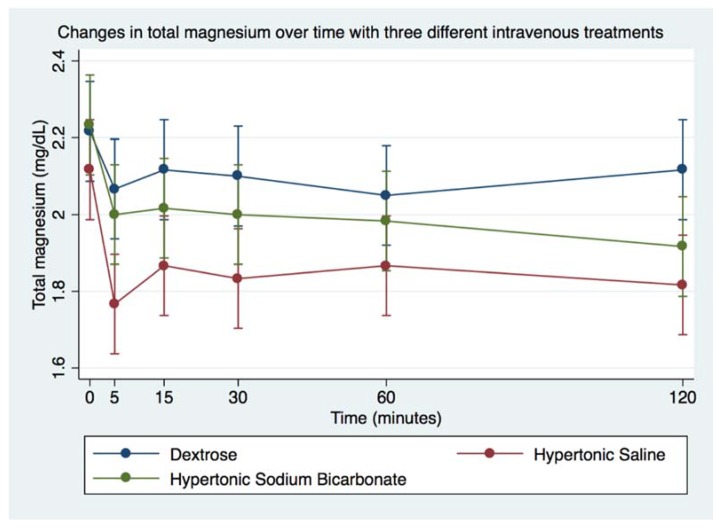
Overall effect of treatment on the blood total magnesium (tMg) over time. The mean and SEM venous blood tMg for all six horses. Blue = Treatment Group DS (5% dextrose solution at 2 mL/kg bwt); Green = Treatment Group HB (NaHCO_3_ 8.4% solution at 2 mmol/kg bwt); and Red = Treatment Group HS (NaCl 7.5% solution at 4 mL/kg bwt).

**Figure 8:**
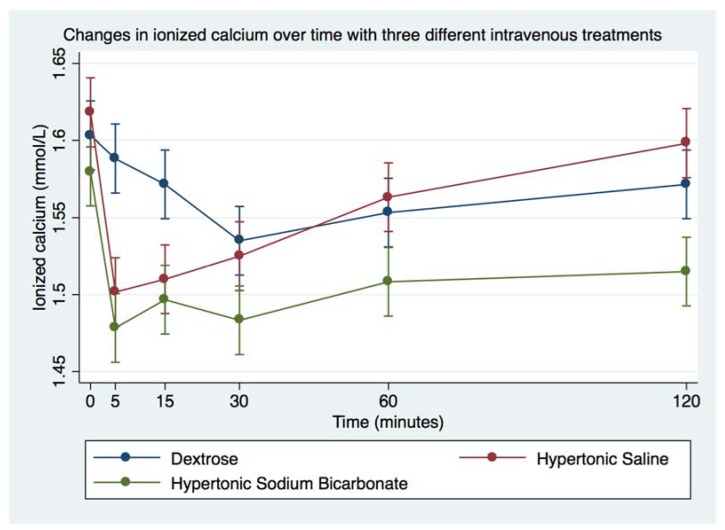
Overall effect of treatment on the blood ionized calcium (Ca^2+^) over time. The mean and SEM venous blood Ca^2+^ for all six horses. Blue = Treatment Group DS (5% dextrose solution at 2 mL/kg bwt); Green = Treatment Group HB (NaHCO_3_ 8.4% solution at 2 mmol/kg bwt); and Red = Treatment Group HS (NaCl 7.5% solution at 4 mL/kg bwt).

Infusion of DS was followed by significant increase in blood glucose and decrease in blood Na^+^. Infusion of HS was followed by significant increase in Na^+^ and Cl^−^, and decrease in Mg^2+^. Infusion of HB was followed by significant decrease in Mg^2+^. Blood tMg, K^+^, and Ca^2+^ did not vary significantly from baseline following any of the treatments, remaining within physiologic ranges, as indicated in Table 5. Magnesium values, especially Mg^2+^, were significantly decreased following infusion of either hypertonic solution (Table 5, Figure 6 and Figure 7). Whereas Ca^2+^ did not go outside of physiologic range with either solution, Table 5, Figure 8).

## 4. Discussion

This study evaluated changes in blood pH and electrolytes following administration of 5% dextrose solution, hypertonic saline, and hypertonic sodium bicarbonate solution with concomitant effect on headshaking behavior in the short term. The minimal effects observed were not expected to last. There was an effect of treatment with HB treatment having a 58% incident rate reduction in median headshakes/minute when compared to DS across all gaits (walk, trot, canter, walk). The reduction was compared to DS treatment which is a solution that is converted to water and would have minimal effect on pH. Despite the fact that there was great variability in the severity of the headshaking behavior between horses and variability of the same horses on different days, the IRR analysis takes into account these variables and provides a way to look at the effect of solution in headshaking behavior. The greatest reduction in headshakes/minute was 67% IRR for the trot with HB treatment. The effect of time at T15 was the first exercise period after receiving intravenous fluid treatment and the cause for that is speculated as the first time to exercise after being restrained for fluid therapy. The increase in rate of headshakes/minute at T60 after HB treatment is speculated as being the effect of intravenous fluids not lasting long, and an hour after administration the effects of the solution were going away. In this study, Thoroughbreds were less severely affected overall, however, they were more sensitive to changes associated with the solutions given. It would be beneficial to increase animal numbers to verify the effect of breed.

Overall, administration of 5% dextrose solution at 2 mL/Kg bwt caused minimal (not significant) increase in pH and a small drop in Na^+^ and Mg^2+^. The mild drop in Na^+^ is likely a result of the dilution effect of the infused solution. The mild drop in Mg^2+^ could be explained by a glucose-mediated increase in transcellular transport of magnesium into the cells leading to a slight decrease in plasma Mg^2+^; additionally, mild diuresis associated with the transient hyperglycemia and presumed glucosuria could have occurred.

Administration of hypertonic saline (8 times the physiologic concentrations of Na^+^ and Cl^−^ in blood) at 4 mL/Kg bwt caused mild decrease in blood pH (remaining within normal ranges), mild decrease in SBE, AG, and SID from baseline, mild hypernatremia and marked hyperchloremia, mild drop in Ca^2+^ (although remaining within reference range), and mild hypomagnesemia (characterized by decrease in Mg^2+^ below normal range and mild decrease in tMg). These changes are likely associated with Na^+^ and Cl^−^ overload, leading to potent volume expansion and natriuresis. This explains the persistent and slightly worsening concentrations of the Mg^2+^ up to the last sampling (120 min).

Finally, administration of hypertonic bicarbonate solution (6 times the physiologic concentrations of Na^+^ in blood) at 2 mL/Kg bwt caused moderate increase in blood pH, marked increase in HCO_3_^−^ and SBE (the latter reaching 3 times the baseline concentration), mild decrease in SID, mild decrease in Cl^−^ (although remaining within normal ranges), and mild decrease in AG. Similar to the effect seen after HS, HB led to mild decrease in Ca^2+^ and tMg (although remaining within normal ranges), and hypomagnesemia (characterized by decreased Mg^2+^) that persisted, worsening slightly over time, up to the last sampling (120 min).

The blood results from this study showed transient changes in blood variables, with significant short-lived changes in acid–base status (blood pH, SBE, HCO_3_^−^, SID, and AG) when the horses were infused with HB, and mild changes in acid–base status (blood pH, SBE, SID, and AG) following HS. When horses were infused with DS, there were virtually no changes in acid–base status from baseline. It is expected that the changes in acid–base status are short-lived and quickly corrected [20]. Indeed, by 30 min post infusion of HB, blood pH was back to the reference range. It is surprising that the acidifying effects of hypertonic saline were so minimal. Treatment with either hypertonic solution led to a decrease in Mg^2+^, likely due to volume expansion and natriuresis. Although the changes in pH and electrolytes were modest, these subtle changes could lead to alterations in nerve firing, thus affecting headshaking.

Baseline values for SBE for all horses were above the reference range, and the calculated AG was below reference range at baseline. This finding was most likely due to diet (DCAB of 31 mEq/100 kg). This could have accounted for the mild acidifying effect of HS administration. The baseline measurements for most of the horses showed marginal levels of Mg^2+^ (reference range from UC Davis VMTH Clinical Diagnostic Laboratory Services: 0.47–0.70 mmol/L). This is surprising considering that the horses should have been receiving their magnesium requirement of 13 mg/kg bwt/day, based on hay analysis of their feed [21,22]. Average absorption of magnesium content in feed by horses is 49.5% with a range of 30–60% [21]. Magnesium absorption can also be impaired by fiber and phytate levels; however, phytate levels were not measured in the hay [22]. It is expected that alkalemia would reduce Mg^2+^ and Ca^2+^ [23,24], but the decrease in Mg^2+^ and Ca^2+^ following HS and HB were comparable. This is likely a result of the overpowering effect of volume expansion and natriuresis of both solutions. One could speculate that decrease in Ca^2+^ would result in less activation of the trigeminal nerve into firing. Greater concentrations of ionized calcium in the blood mean that they can activate release of neurotransmitters to signal the nerve to fire [25]. It has been shown that calcium does affect neuropathic pain in the trigeminal nerve [18]. The effects of calcium and magnesium on neuropathic pain should be further researched.

The main limitations of the current study included natural disease having a broad range of clinical signs and severity, the small group of affected horses, and lack of control groups (affected but untreated group and unaffected group). The observed changes in headshaking behavior following the infusion of the different solutions to this group of horses demonstrated a relative decrease in headshaking following HB treatment. A larger group of affected horses might be needed to fully investigate these effects, changes in pH and electrolytes, in such an impairing disorder. Further studies are warranted to establish if dietary changes, especially with respect to calcium and magnesium, affect headshaking behavior and trigeminal nerve firing.

## 5. Conclusions

The pH and electrolyte changes associated with hypertonic sodium bicarbonate solution decreased headshaking behavior in this group of horses affected with trigeminal mediated headshaking. 

## Figures and Tables

**Table 1:** Individual headshaking behaviors at the trot and canter across all times and treatments. Median and IQR for the headshakes/minute. ∆ signifies the change from T0 to T120. * signifies a *p* < 0.05 and ** signifies a *p* < 0.01.

**DS**												
**Trot**												
	**T0**		**T15**		**T30**		**T60**		**T120**		**Overall**	
**Horse ID**	**Median**	**IQR**	**Median**	**IQR**	**Median**	**IQR**	**Median**	**IQR**	**Median**	**IQR**	**Range**	**∆**
1	11	3	2 *	2	8	0.5	7	1	10	3	2 to 11	−1
2	1	1	4	1	3	1	5	2	1	0	1 to 5	0
3	0	1	1	0	0	0	3	2	0	0	0 to 3	0
4	5	2	10	3	10	1	6	6	7	4	5 to 10	2
5	3	2	5	3	5	0	3	2	11 *	2	3 to 11	8
6	0	0	0	0	0	0	0	0	1	0	0 to 1	1
**HS**												
**Trot**												
	**T0**		**T15**		**T30**		**T60**		**T120**		**Overall**	
**Horse ID**	**Median**	**IQR**	**Median**	**IQR**	**Median**	**IQR**	**Median**	**IQR**	**Median**	**IQR**	**Range**	**∆**
1	9	2	15	2	7	2	5	2	5	2	5 to 15	−4
2	1	1	0	0	1	0	1	0	0	0	0 to 1	−1
3	0	0	1	0	1	0	2	1	0	0	0 to 2	0
4	6	3	5	5	5	2	6	2	4	1	4 to 6	−2
5	7	0	7	3	10	1	1	1	3	1	1 to 10	−4
6	0	0	5	2	9	2	3	2	1	0	0 to 9	1
**HB**												
**Trot**												
	**T0**		**T15**		**T30**		**T60**		**T120**		**Overall**	
**Horse ID**	**Median**	**IQR**	**Median**	**IQR**	**Median**	**IQR**	**Median**	**IQR**	**Median**	**IQR**	**Range**	**∆**
1	6	2	11	3	12	3	7	6	8	2	6 to 12	2
2	2	1	1	1	1	1	1	0	1	0	1 to 2	−1
3	0	0	0	0	0	0	0	0	0	0	0 to 0	0
4	1	1	1	0	2	2	1	1	1	1	1 to 2	0
5	7	1	5	1	5	2	3	1	2	1	2 to 7	−5
6	0	0	3	1	1	0	0	1	0	0	0 to 3	0
**DS**												
**Canter**												
	**T0**		**T15**		**T30**		**T60**		**T120**		**Overall**	
**Horse ID**	**Median**	**IQR**	**Median**	**IQR**	**Median**	**IQR**	**Median**	**IQR**	**Median**	**IQR**	**Range**	**∆**
1	26	21	22	2	29	20	27	6	33	6	22 to 33	7
2	1	1	1	1	2	3	5	1	0	0	0 to 5	−1
3	0	0	0	0	1	0	1	1	0	0	0 to 1	0
4	14	10	20	22	16	16	8	3	11	3	8 to 20	−3
5	9	1	6	3	6	3	10	4	17	4	6 to 17	8
6	3	1	1	0	1	1	2	1	2	2	1 to 3	−1
**HS**												
**Canter**												
	**T0**		**T15**		**T30**		**T60**		**T120**		**Overall**	
**Horse ID**	**Median**	**IQR**	**Median**	**IQR**	**Median**	**IQR**	**Median**	**IQR**	**Median**	**IQR**	**Range**	**∆**
1	23	4	52 **	4	24	6	25	9	16	4	16 to 52	−7
2	0	0	0	1	2	1	2	0	4	3	0 to 4	4
3	2	0	0	0	1	1	0	0	3	1	0 to 3	1
4	12	3	5	2	4	3	5	0	6	1	4 to 12	−6
5	6	2	6	4	6	2	1	2	4	3	1 to 6	−2
6	2	1	12 *	3	6	4	3	3	2	0	2 to 12	0
**HB**												
**Canter**												
	**T0**		**T15**		**T30**		**T60**		**T120**		**Overall**	
**Horse ID**	**Median**	**IQR**	**Median**	**IQR**	**Median**	**IQR**	**Median**	**IQR**	**Median**	**IQR**	**Range**	**∆**
1	8	5	12	16	19 *	15	29 **	9	22 *	6	8 to 29	14
2	2	1	0	0	0	0	4	1	2	3	0 to 4	0
3	0	0	0	0	0	0	0	1	0	0	0 to 0	0
4	2	1	0	1	2	2	3	0	5	8	0 to 5	3
5	7	4	8	5	4	4	3	3	3	2	3 to 8	−4
6	1	0	3	3	1	1	1	1	0	0	0 to 3	−1

**Table 2:** Effect of treatment and period on incidence rate ratio (IRR) of median headshakes/minute across all times. IRR, *p* value and confidence intervals. *p* < 0.05 was considered significant.

Treatment	IRR	*p* Value	C.I. 95%	
HS				
All periods	0.95	0.488	0.83	1.09
Trot	0.98	0.898	0.76	1.27
Canter	0.86	0.099	0.72	1.02
HB				
All periods	0.58	0.000	0.49	0.67
Trot	0.67	0.005	0.51	0.89
Canter	0.52	0.000	0.43	0.64

**Table 3:** Effect of treatment and time on incidence rate ratio (IRR) of median headshakes/minute at the trot and canter. IRR, *p* value and confidence intervals. *p* < 0.05 was considered significant.

Behavior	Treatment											
	DS				HS				HS			
	IRR	*p*	C.I. 95%		IRR	*p*	C.I. 95%		IRR	*p*	C.I. 95%	
Headshakes												
Trot												
T 15 min	1.10	0.758	0.6	2.02	1.43	0.184	0.84	2.44	1.31	0.413	0.68	2.51
T 30	1.30	0.378	0.72	2.32	1.43	0.184	0.84	2.44	1.31	0.413	0.68	2.51
T 60	1.20	0.547	0.66	2.17	0.78	0.436	0.42	1.45	0.75	0.451	0.35	1.59
T120	1.50	0.160	0.85	2.64	0.57	0.100	0.29	1.11	0.75	0.451	0.35	1.59
Canter												
T 15 min	0.94	0.768	0.64	1.38	1.67	0.007	1.15	2.41	1.15	0.648	0.63	2.09
T 30	0.96	0.845	0.66	1.41	0.93	0.748	0.61	1.42	1.30	0.378	0.72	2.32
T 60	1.00	1.000	0.68	1.46	0.80	0.318	0.52	1.24	2.00	0.011	1.17	3.42
T120	1.18	0.354	0.82	1.71	0.78	0.265	0.5	1.21	1.60	0.099	0.92	2.80

**Table 4:** Effect of treatment and breed on incidence rate ratio (IRR) of median headshakes/minute at the trot and canter and all periods. IRR, *p* value and confidence intervals. *p* < 0.05 was considered significant.

Treatment	Quarter Horse Breeds				Thoroughbreds			
	IRR	*p* Value	C.I. 95%		IRR	*p* Value	C.I. 95%	
HS								
All periods	0.82	0.007	0.70	0.95	2.83	<0.001	1.85	4.31
Trot	0.84	0.196	0.64	1.09	4.40	0.003	1.66	11.61
Canter	0.78	0.008	0.64	0.94	2.81	0.003	1.42	5.60
HB								
All periods	0.57	<0.001	0.49	0.67	0.69	0.201	0.39	1.22
Trot	0.66	0.006	0.50	0.88	0.80	0.156	0.15	1.35
Canter	0.52	<0.001	0.42	0.64	0.55	0.232	0.20	1.47

**Table 5:** Blood Analysis Variable. Changes in blood variables following intravenous infusion of DS, HB, and HS. DS (5% dextrose solution at 2 mL/kg bwt), HB (NaHCO_3_ 8.4% solution at 2 mmol/kg bwt), and HS (NaCl 7.5% solution at 4 mL/kg bwt). Numbers in bold for the following parameters: pH, HCO_3_, Na^+^, Cl^−^, K^+^, glucose, Ca^2+^, tMg, and Mg^2+^ indicate values above or below the reference range for that blood parameter. Note: For SBE, the baseline values for all horses were above the reference range. For this parameter, bold indicates difference from baseline values.

Blood Parameter	Treatments					
	DS		HS		HB	
	Mean	SEM	Mean	SEM	Mean	SEM
pH (ref 7.35 to 7.45)						
0	7.375	±0.004	7.370	±0.003	7.381	±0.005
5	7.402	±0.003	7.359	±0.003	7.479	±0.003
15	7.388	±0.003	7.368	±0.003	7.451	±0.002
30	7.403	±0.004	7.366	±0.002	7.457	±0.003
60	7.425	±0.003	7.389	±0.002	7.436	±0.003
120	7.413	±0.004	7.376	±0.004	7.439	±0.005
SBE mmol/L (ref −3.0 to +3.0)						
0	6.1	±0.3	4.8	±0.2	5.4	±0.3
5	5.7	±0.3	2.3	±0.2	14.0	±0.4
15	6.0	±0.3	3.3	±0.2	12.6	±0.4
30	6.5	±0.3	4.1	±0.2	11.9	±0.4
60	7.1	±0.4	4.4	±0.2	10.6	±0.4
120	6.5	±0.3	3.0	±0.3	9.1	±0.3
HCO_3_^−^ mmol/L (ref 25 to 32)						
0	31.1	±0.3	29.8	±0.2	30.3	±0.3
5	30.4	±0.3	27.4	±0.2	38.6	±0.5
15	30.9	±0.2	28.2	±0.2	37.3	±0.4
30	31.3	±0.3	29.1	±0.3	36.5	±0.4
60	31.5	±0.4	29.1	±0.3	35.2	±0.4
120	31.1	±0.3	27.8	±0.3	33.6	±0.3
Na^+^ mEq/L (ref 135 to 145)						
0	138	±0.4	136	±0.4	137	±0.3
5	134	±0.4	146	±0.4	139	±0.2
15	135	±0.4	145	±0.4	139	±0.1
30	136	±0.5	144	±0.2	139	±0.2
60	136	±0.5	143	±0.4	136	±0.3
120	136	±0.6	138	±0.6	136	±0.2
Cl^−^ mEq/L (ref 94 to 102)						
0	103	±0.3	103	±0.4	102	±0.3
5	101	±0.3	118	±0.4	99	±0.3
15	102	±0.4	115	±0.4	99	±0.3
30	103	±0.5	114	±0.4	100	±0.3
60	103	±0.5	113	±0.4	99	±0.4
120	102	±0.6	108	±0.5	99	±0.4
K^+^ mEq/L (ref 3.3 to 5.0)						
0.0	4.4	±0.2	4.4	±0.1	4.0	±0.2
5.0	4.9	±0.1	4.2	±0.0	4.4	±0.1
15.0	4.8	±0.2	4.4	±0.1	4.4	±0.2
30.0	5.0	±0.2	4.4	±0.1	4.3	±0.1
60.0	4.8	±0.1	4.3	±0.1	4.0	±0.1
120.0	4.3	±0.1	3.7	±0.1	3.5	±0.1
Glucose mg/dL (ref 77 to 110)						
0	91	±2	91	±2	96	±0
5	156	±3	86	±1	99	±1
15	134	±3	88	±1	100	±1
30	98	±3	92	±1	82	±4
60	89	±1	96	±0	92	±1
120	92	±1	95	±1	93	±1
Lactate mmol/L (ref < 2 )						
0	0.8	±0.0	1.0	±0.1	0.8	±0.1
5	1.0	±0.0	0.6	±0.1	0.9	±0.1
15	1.1	±0.1	0.6	±0.0	1.0	±0.1
30	0.9	±0.0	0.6	±0.0	0.7	±0.1
60	0.9	±0.1	0.6	±0.0	0.8	±0.1
120	1.1	±0.1	0.7	±0.1	0.8	±0.0
Ca^2+^ mmol/L (ref 1.40 to 1.60)						
0	1.60	±0.01	1.62	±0.00	1.58	±0.01
5	1.59	±0.01	1.50	±0.01	1.48	±0.01
15	1.57	±0.01	1.51	±0.01	1.50	±0.01
30	1.54	±0.01	1.53	±0.01	1.48	±0.00
60	1.55	±0.01	1.56	±0.01	1.51	±0.00
120	1.57	±0.01	1.60	±0.01	1.52	±0.01
tMg mg/dL (ref 1.9 to 3.0)						
0	2.2	±0.03	2.1	±0.04	2.2	±0.05
5	2.1	±0.03	1.8	±0.03	2.0	±0.04
15	2.1	±0.03	1.9	±0.04	2.0	±0.03
30	2.1	±0.03	1.8	±0.04	2.0	±0.03
60	2.1	±0.02	1.9	±0.04	2.0	±0.02
120	2.1	±0.03	1.8	±0.04	1.9	±0.02
Mg^2+^ mmol/L (ref 0.47 to 0.70)						
0	0.51	±0.02	0.44	±0.01	0.46	±0.01
5	0.50	±0.02	0.40	±0.01	0.40	±0.01
15	0.49	±0.02	0.40	±0.01	0.41	±0.01
30	0.48	±0.02	0.40	±0.01	0.41	±0.01
60	0.47	±0.02	0.39	±0.01	0.40	±0.01
120	0.50	±0.01	0.39	±0.01	0.39	±0.00

**Table 6:** Calculated Blood Results. Calculated changes in blood variables following intravenous infusion of DS, HB, and HS. DS (5% dextrose solution at 2 mL/kg bwt), HB (NaHCO_3_ 8.4% solution at 2 mmol/kg bwt), and HS (NaCl 7.5% solution at 4 mL/kg bwt). Numbers in bold for the following indicated values above or below the reference range for that blood parameter.

Blood Parameter	Treatments					
	DS		HS		HB	
	Mean	SEM	Mean	SEM	Mean	SEM
SID mmol/L (ref 38–42)						
0	41	±0	40	±0	41	±0
5	40	±0	**35**	**±0**	**46**	**±0**
15	40	±0	**36**	**±0**	**46**	**±0**
30	40	±0	**36**	**±0**	**45**	**±0**
60	40	±0	**36**	**±0**	**43**	**±0**
120	40	±0	**35**	**±0**	42	±0
AG mmol/L (ref 9–17)						
0	**8**	**±0**	**8**	**±0**	**8**	**±1**
5	**7**	**±0**	**6**	**±0**	**6**	**±0**
15	**7**	**±0**	**6**	**±0**	**7**	**±0**
30	**7**	**±0**	**5**	**±0**	**6**	**±0**
60	**7**	**±0**	**5**	**±0**	**6**	**±0**
120	**7**	**±0**	**6**	**±0**	**7**	**±0**
